# COVID-19 Vaccine Hesitancy in Healthcare Personnel: A University Hospital Experience

**DOI:** 10.3390/vaccines9111343

**Published:** 2021-11-17

**Authors:** Beril Kara Esen, Gunay Can, Betul Zehra Pirdal, Sumeyye Nur Aydin, Aysenur Ozdil, Ilker Inanc Balkan, Beyhan Budak, Yilmaz Keskindemirci, Ridvan Karaali, Nese Saltoglu

**Affiliations:** 1 Department of Public Health, Cerrahpasa Faculty of Medicine, Istanbul University-Cerrahpasa, Kocamustafapasa, Fatih, Istanbul 34098, Turkey; gunay.can@iuc.edu.tr (G.C.); zehrapirdal@istanbul.edu.tr (B.Z.P.); nur.aydin@istanbul.edu.tr (S.N.A.); aysenur.ozdil@istanbul.edu.tr (A.O.); 2 Department of Infectious Diseases, Cerrahpasa Faculty of Medicine, Istanbul University-Cerrahpasa, Kocamustafapasa, Fatih, Istanbul 34098, Turkey; ilker.balkan@istanbul.edu.tr (I.I.B.); beyhan.budak@iuc.edu.tr (B.B.); ridvan.karaali@iuc.edu.tr (R.K.); saltoglu@iuc.edu.tr (N.S.); 3 Vocational School of Health Services, Department of Medical Services and Techniques, Cerrahpasa Faculty of Medicine, Istanbul University-Cerrahpasa, Kocamustafapasa, Fatih, Istanbul 34098, Turkey; kdemirci@istanbul.edu.tr

**Keywords:** COVID-19, vaccination, healthcare personnel, vaccine hesitancy

## Abstract

Healthcare workers are among risk groups in the COVID-19. Even if they are not infected with the disease, they witness the effects of the pandemic. The aim of the study is to determine the factors affecting COVID-19 vaccination status and reasons for vaccine hesitancy of healthcare personnel in our hospital. Firstly, the vaccination status and demographic characteristics of all healthcare personnel was evaluated. After that, a survey was applied to 408 vaccinated and 297 nonvaccinated personnel. Within the first month after the beginning of vaccination, 66% of 3937 healthcare personnel received a COVID-19 vaccine. The number of vaccinated personnel was higher among doctors, master graduates or higher educational levels and basic science-laboratory unit workers. In the surveyed group, being under the age of 50 (OR:1.85), being nondoctor healthcare personnel (nurse/midwife OR:1.78, administrative personnel OR:3.42, patient attendant/cleaning staff OR:4.11, security guard/other OR:2.96), having had the disease before (OR:2.36), not having the flu vaccine (OR:3.24) and hesitancy about other vaccines (OR:6.61) were found to be independent risk factors for not having a COVID-19 vaccine or having it late. The three most common reasons for not getting vaccinated were doubt on the efficacy of the vaccine, distrust of its content, and fear of side effects. Taking steps by considering the main factors of hesitancy among healthcare personnel will increase the vaccine acceptance.

## 1. Introduction

Following the initial announcement that a cluster of new viral pneumonia cases were identified in China on 31 December 2019, similar pneumonia cases began to be reported rapidly by different countries in 2020. The illness, recognized as being caused by a new strain of the coronavirus family, was called COVID-19 disease by the World Health Organization (WHO) on 11 February 2020 and declared a pandemic on 11 March 2020 [1].

It is known that people with underlying comorbidities and the elderly are especially at risk of severe illness from COVID-19, occupational groups working in close contact with patients and other people are also at high risk of contracting the disease [2]. Healthcare workers are considered amongst ‘very high risk’ occupational groups by Occupational Safety and Health Administration (OSHA) [3].

During the fight against the disease, mainly transmitted by droplets, protective public health measures such as masks, social distancing and good hygiene were primarily utilized. In addition, the COVID-19 disease was tackled through various medical treatments administered by researchers and doctors worldwide. Whilst treatment methods continue to be explored for the illness, yet with no definitive cure, vaccine studies, with vaccines as one of the most important inventions of humankind, were initiated worldwide. The first emergency use authorization for a vaccine developed against the disease was issued by the Food and Drug Administration (FDA) on 11 December 2020 [4] and the chief step taken in vaccination of the masses. As of August 2021, a total of seven different COVID-19 vaccines having approval for emergency use are widely administered around the world [5].

Along with the report at the WHO General Assembly held on 24 May 2021 that at least 115 thousand health workers had died due to COVID-19 since the beginning of the pandemic, 403 healthcare workers had also lost their lives in Turkey by 31 May 2021 [6,7]. Whilst health workers are at an increased risk of acquiring the COVID-19 disease, they are the occupational group witnessing the devastating effects of the pandemic most, even if they do not become infected themselves. As a result, they are expected to comply with the protective measures and to be a role model for other people.

In Turkey, as of 14 January 2021, administration of vaccines against COVID-19 started with the inactive COVID-19 vaccine (developed by the Sinovac company), and healthcare personnel, being part of the group with priority and at risk, were the first to be vaccinated. Though there were 2,364,801 COVID-19 cases in Turkey as of 14 January 2021, 23,495 deaths occurred [8]. However, with the introduction of vaccinations, hesitancy against COVID-19 vaccines for various reasons was also observed amongst health workers, some refusing or delaying vaccination against COVID-19. As vaccine hesitancy against vaccinations that have been in use for many years has reached a serious level, it is an expected situation that a new vaccine developed against a new disease such as COVID-19 would be approached with uncertainty.

As of 31 March 2021, upon the arrival of a vaccine produced with mRNA technology (developed by Pfizer–BioNTech), two types of COVID-19 vaccines have been introduced for administration in Turkey, and there are no restrictions on the choice of the vaccine. With the recommendation of the World Health Organization and the ministry, for those who have had the first dose of vaccine, a rule exists to administer the same type for the second dose of vaccine.

Our study aimed to determine the factors affecting the COVID-19 vaccination status of health personnel and their reasons for not receiving it. The study was conducted at a university hospital which is one of the well-established institutions in the field of medical education and healthcare in Turkey, where 258,353 tests were performed between 10 March 2020 and 30 August 2021 with 25,818 positive test results (test positivity rate 10%), providing care to more than 124 thousand outpatients, 3652 patients admitted in pandemic wards and 738 patients followed up in intensive care units.

## 2. Materials and Methods

Healthcare personnel working at Istanbul University-Cerrahpasa Faculty of Medicine participated in the study.

### 2.1. Analysis of All Staff

**Figure 1:**
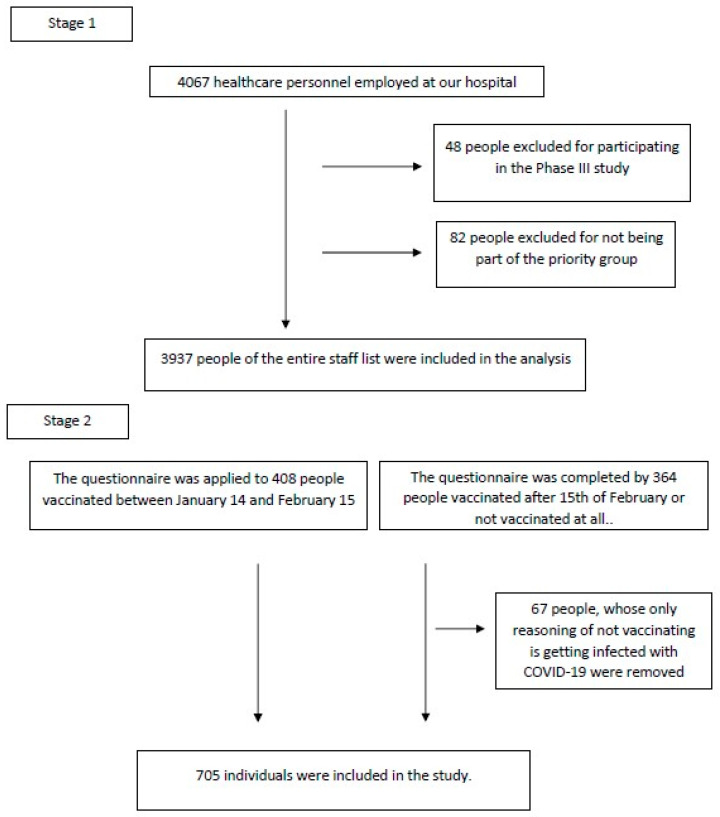
Selection of participants.

Whilst the age, gender, unit, title and education level of all personnel working and recorded in our hospital were obtained from the list of registered personnel in the hospital, the vaccination status of individuals, date of vaccination, what type of vaccine they had and the number of doses received were gathered from the data in our hospital’s vaccination outpatient clinic. Vaccination information of people vaccinated in another center was withdrawn from the ‘Vaccinate’ application. These demographic characteristics and vaccination status were evaluated retrospectively. As of July 4, the date of data collection, out of 4067 personnel registered in our hospital the following groups were excluded from analysis: forty-eight people for participating in the phase 3 study and eighty-two people for not being in the priority group (Figure 1).

### 2.2. Personnel Surveyed

To evaluate the factors that may be associated with not having the vaccination against COVID-19, it was aimed to have the questionnaire prepared by our unit completed by all staff. Whilst healthcare personnel receiving the vaccine between January 14 and February 15 constituted the control group, those who had the first dose after February 15 or did not have it at all were included in the unvaccinated group. It was planned to reach all personnel within two weeks after March 17. Prior to administering the questionnaire, an informed consent form for the study was given to the individuals contacted. Included in this form was general information about the study, by whom it was carried out, and informing that the survey data would be used for the purpose of the study with confidentiality. The questionnaire was applied to those who agreed to participate in the study and signed the consent form. Personnel refusing to take part in the study, those who did not sign the informed consent form, employees working at another center because of a temporary assignment and people not employed in our hospital were not included. Four hundred eight people formed the group who had the first dose of vaccine within the specified time period; 298 people who were not vaccinated or had it late completed the survey. As the right to vaccination for health personnel was established one month after acquiring COVID-19, 67 people within the unvaccinated group were excluded from the analysis for not having the vaccine only due to previous infection (Figure 1).

The participants of the study were contacted individually and the questionnaire consisting of 20 questions was conducted in-person. By the questions within the survey, it is intended to determine sociodemographic characteristics (8 questions), having increased risk in terms of COVID-19 (3 questions), the case of self or a relative acquiring COVID-19 previously (2 questions), circumstance of fearing the COVID-19 disease (1 question), attitude towards other vaccines (3 questions), self-perception of health (2 questions) and hesitancy regarding previous vaccines (2 questions). For healthcare personnel deferring or refusing the vaccination, a question was also included in relation to the reason for this delay or refusal. An informed consent form was signed by the participants.

For our research, ethical committee approval (dated 17 March 2021 and numbered 53843) was obtained from the Istanbul University-Cerrahpasa Medical Faculty Clinical Research Ethics Committee.

### 2.3. Statistical Analysis

Statistical analysis was performed using SPSS version 21. In descriptive analyses, the number and percentage in categorical variables, mean ± standard deviation or median, 25–75th percentiles or minimum–maximum values were given where appropriate. Normality of the continuous variables was demonstrated via the Kolmogorov–Smirnov test, coefficient of variation, histogram and Q–Q plot. Comparisons between the groups were evaluated with Mann–Whitney *U* test for continuous variables and with chi-squared test or Fisher’s exact test for categorical variables. In comparisons found significant between more than two groups in categorical variables, it was determined using the post hoc Bonferroni test between which groups the significance lies. To identify the factors that increase the risk of delaying or not having the vaccination, variables with a *p*-value below 0.250 in univariate analysis were assessed with binary logistic regression analysis. Backward LR method was used in regression analysis. A *p*-value below 0.05 was considered significant.

## 3. Results

### 3.1. Evaluation of All Personnel Working at the Hospital in Terms of Demographic Characteristics

Amongst the 3937 people on the general hospital staff list, there were 3299 people who had at least 1 dose of a COVID-19 vaccine by the 4th of July; the number of people not receiving any COVID-19 vaccine was 638. Comparing the two groups with and without vaccination, the median age of the people in the vaccinated group (38 (30–48)) was found to be significantly higher than the median age (33 (27–41)) of the people in the unvaccinated group (*p* < 0.001). There was no meaningful difference between the genders in terms of vaccination status (*p* = 0.385). Out of staff members in our faculty, the vaccination rate was detected to be 90.5% among those who have a master graduates/medical specialization or are at doctoral level, 80.4% among from university graduates, 77.6% among high school graduates, 78.5% among secondary school graduates and 87% among primary school graduates. In this case, the vaccination rate of the personnel in the group with the highest education level was not different from that of primary school graduates, but it was proven to be significantly higher than those of university, high school and secondary school graduates (*p* < 0.001). When examining the occupational groups of the personnel, the rate of vaccination was 91% in doctors, 78.1% in nurses, 84% in health-related technicians, 81.7% in administrative staff and 82.4% in the cleaning personnel/patient attendant group. The vaccination rate amongst doctors was demonstrated to be significantly higher than all other occupational groups (<0.001). Examining the employees in terms of the unit they were assigned to, the vaccination rate of the basic sciences and laboratory personnel was 91.6%, 83.1% in the internal medicine personnel, 84.5% in the surgical personnel and 80% in administrative–technical team. The vaccination rate of the personnel working in basic sciences and laboratories was revealed to be considerably higher than those employed in other units (<0.001) (Table 1).

By the 4th of July 2021, 2975 (90.2%) of 3299 people were immunized with the inactivated vaccine and 324 (9.8%) people were given the mRNA vaccine. Considering the number of doses administered, 3029 people (91.8%) were vaccinated with two doses, yet 270 people (8.2%) received a single dose of vaccine.

As of 31 March 2021, two types of vaccines had been introduced in the country. Following this date, 126 people were given the inactivated COVID-19 vaccine while 324 people were vaccinated with mRNA vaccine. In review of whether there was a difference between the two vaccine types regarding demographic characteristics of the individuals, no relationship was found between being under or over the age of 50 and the type of the vaccine (*p* = 0.305). There was no significant difference between people in the two vaccination groups relating to educational status or title (*p* = 0.244 and *p* = 0.088, respectively). Upon evaluating the individuals with the two vaccine types in terms of the unit in which they work, only the rate of those working in the surgical units among individuals given the mRNA vaccine was detected to be significantly higher in comparison to the rate of those employed in the surgical units in the inactivated vaccine group (*p* = 0.041).

### 3.2. Comparison of Vaccinated and Unvaccinated People Participating in the Questionnaire

**Figure 2:**
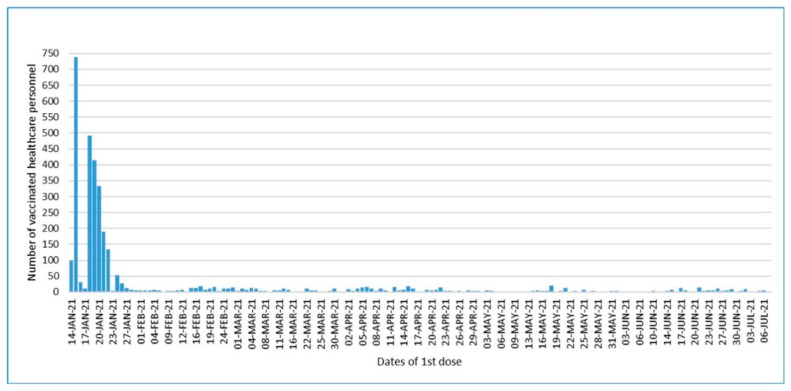
Dates of first vaccine dose in the vaccinated personnel.

As of 15 February 2021, following the beginning of vaccination, 66% of our personnel received a COVID-19 vaccine (Figure 2). A survey was conducted by contacting 408 people who were vaccinated and to 297 people who had a vaccination after February 15 or were not immunized at all.

In assessing the vaccinated and unvaccinated groups in terms of demographic characteristics, it was recognized that the rate of people under the age of 50 in the unvaccinated group was 89.5% whilst 83.7% in the vaccinated group. The proportion of people under age 50 in the unvaccinated group was significantly higher (*p* = 0.033). Evaluating in terms of gender, no significant difference was found between the groups that had or did not have the vaccine (*p* = 0.210). There was no meaningful distinction between the vaccinated and unvaccinated groups regarding marital and childbearing status (*p* = 0.603 and *p* = 0.157). When the participants in both groups were compared in terms of education levels, the rate of those who were masters or higher degree graduates in the vaccinated group (42.5%) was detected to be remarkably higher than that of the unvaccinated group (21.9%), whereas the proportion of university and high school graduates was higher in the unvaccinated group (*p* < 0.001). There was no difference between the vaccinated and unvaccinated groups in regard to being a primary or secondary school graduate. Reviewing both groups in relation to occupational groups of the respondents, the rate of physicians/lecturers in the vaccinated group (34.8%) was revealed to be considerably greater than that of the unvaccinated group (16.2%), yet the rate of administrative staff, patient attendant/cleaning staff and security guards/other category was significantly higher in the unvaccinated group (*p* < 0.001). There was no significant difference between the two groups regarding the rate of nurse/midwife (Table 2).

As the rate of those with a chronic disease amongst people in the vaccinated group was 32.1%, in the unvaccinated group was 24.9% (*p* < 0.038). There was no significant difference between the two groups in terms of living with/caring for someone who is at risk of COVID-19, working in departments providing one-to-one care to a COVID-19 patient and the condition of the ones with COVID-19 disease in their family or close circle (*p* = 0.342, *p* = 0.112 and *p* = 0.824, respectively). The rate of people infected with COVID-19 in the unvaccinated group (30.7%) was shown to be significantly higher than the rate of people who had COVID-19 in the vaccinated group (18.2%) (*p* < 0.001). As the individuals in both groups were compared regarding the fear of contracting COVID-19 disease, there was no remarkable difference between the two groups (*p* = 0.307) (Table 2).

There was no significant difference between the two groups in terms of the participants’ perceptions regarding their own health status and of their health condition this year compared to last year (*p* = 0.541 and *p* = 0.304, respectively) (Table 2).

In consideration of the questions presented to the participants concerning their attitudes toward other vaccines, the rate of those in the vaccinated group (81.7%) receiving hepatitis A, hepatitis B or tetanus vaccines, which are recommended vaccines for healthcare workers, was found to be significantly higher than the rate of those who had one of these vaccines in the unvaccinated group (73.5%) (*p* = 0.009). There was no remarkable difference between the vaccinated and unvaccinated groups in terms of the proportion of people who previously had a paid vaccination for themselves or their children (*p* = 0.244). The rate of those who had the flu vaccine this year was 22.9% in the vaccinated group, while it was 8.8% in the unvaccinated group (*p* < 0.001) (Table 2).

In review of the questions asked to individuals relating to the hesitancy concerning other vaccines, the rate of those who refused a vaccine to be given to themselves or their children due to believing them to be dangerous or unhelpful was revealed to be remarkably greater in the group rejecting a COVID-19 vaccine (22.1%) compared to the group having a COVID-19 vaccine (2.9%) (*p* < 0.001). The rate of those who previously postponed a vaccine recommended by a doctor was also significantly higher in the group who rejected a COVID-19 vaccine (12.5%) compared to those who had a COVID-19 vaccine (2.9%) (*p* < 0.001). In an evaluation conducted by regarding the individuals answering yes to one of these two questions as vaccine hesitant, the rate of those who were hesitant in terms of other vaccines was found to be higher in the group not receiving a COVID-19 vaccine (29.6%) in contrast to the group vaccinated (5.1%) (*p* < 0.001) (Table 2).

Variables that may have an impact on not getting vaccinated or delaying it were assessed using multivariate logistic regression analysis. According to the regression result, it was demonstrated that being under the age of 50 increased the risk of not having a COVID-19 vaccine or deferring it by 1.85 times (95% CI: 1.08–3.18, *p* = 0.026). Gender was not identified to be a risk factor (*p* = 0.209). In comparison to the faculty members or doctors, the risk of not receiving a vaccine or postponement was detected to be higher by 1.78 times for nurses/midwives, by 3.42 times for administrative staff, 4.11 times for patient attendants/cleaners and by 2.96 times for those in the security guard/other group (*p* < 0.001). Suffering from COVID-19 disease compared to not acquiring it elevated the risk of not being immunized with a COVID-19 vaccine or delaying it by 2.36 times (*p* < 0.001). In contrast to those receiving the flu vaccine this year, those not receiving the flu vaccine were observed to have an increased risk of not taking a COVID-19 vaccine by 3.24 times (95% CI: 1.90–5.55, *p* < 0.001). It was revealed that the risk of not getting immunized with a COVID-19 vaccine or late administration was 6.61 times greater for those with vaccine hesitancy related to other vaccines (95% CI: 3.82–11.44, *p* < 0.001). In another regression model we created, adding separately the two questions measuring hesitancy for prior vaccines, it was shown that refusing a vaccine to be given to oneself or one’s child considering it dangerous or ineffective resulted in not vaccinating or deferment with a 7.09 times greater risk, whereas delaying a vaccine previously recommended by the doctor led to an increased risk of 3.34 times (*p* < 0.001 and *p* = 0.003). The risk factors of the previous model were present in this model as well (Table 3).

**Figure 3:**
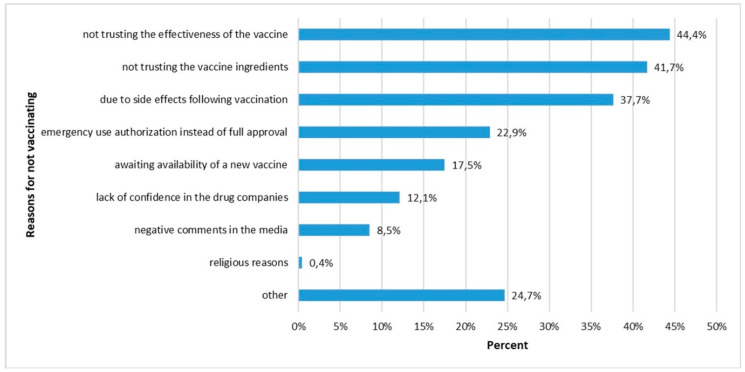
Reasons for not vaccinating that were indicated by the unvaccinated healthcare personnel.

Out of 297 respondents taking the questionnaire who were unvaccinated or received it late, 223 people replied to the question regarding their reasons. In reviewing the reasons among the participants for not getting immunized, it was found that the three most common reasons were not having confidence in the effectiveness of the vaccine, not trusting the vaccine composition and being afraid of the side effects. The fact that the vaccine was authorized for emergency use instead of a full approval was mentioned by 51 people as a justification for not getting vaccinated. At that time, only the inactivated vaccine was used in Turkey and 39 people stated that they were waiting for another vaccine to arrive. Whilst distrust of pharmaceutical companies and negative reviews in the media were the next reasons for vaccine rejection, one individual declared religious reasons as the basis for refusal (Figure 3). Amongst 55 people who marked the other option as the reason for not having the vaccine, it was noted that most common refusal reasons in this group the vaccine was rejected by 7 people due to pregnancy, 7 people due to allergy and 4 people because of breastfeeding.

When the follow-ups were reviewed, as of 4th of July, it was observed that 170 personnel vaccinated in the unvaccinated group. Overall, there was no significant difference between the survey questions of 170 people immunized in the late period and of 117 people still unvaccinated. However, analyzing the 218 people who answered the question about the reasoning behind not getting vaccinated, the rate of those waiting for another vaccine to arrive was 25.2% among those who received the vaccine in the late period whereas it was 9.3% amongst those not receiving the vaccine (*p* = 0.002).

## 4. Discussion

Our study contributes to the determination of factors that may be related to the reasons for not vaccinating by evaluating the vaccination status of the health personnel at our hospital within the first month following initiation of vaccination in our country. During this period, only inactivated vaccine was available in our country and there was no obligation to vaccinate. Vaccines were administered by family doctors or at the hospitals appointed by the ministry as vaccination centers. As vaccination was provided initially to only healthcare workers and then to the elderly, there was no issue of stock shortage related to the vaccine in the first one-month vaccination period. It was established that 66% of our personnel had been vaccinated within the first month. This study showed that being under the age of 50, being healthcare personnel other than a doctor, having had the disease before, not having the flu vaccine in the same year and hesitancy concerning other vaccines, were found to be independent risk factors for not having a COVID-19 vaccine or having it late. Our findings have some similarities and differences to prior studies.

In our study, consistent with the literature, it was shown that the vaccinated personnel were older in comparison to the unvaccinated group, and the group under the age of 50 had 1.81 times more risk of not being vaccinated or having it late. In a systematic review of COVID-19 vaccine hesitancy amongst healthcare workers, it was demonstrated in more than half of the studies examined that people in older age groups had a greater rate of COVID-19 immunization from a vaccine [9]. In a study conducted in Turkey, it was observed that those aged 40–49 or those aged 50 and over were more willing to receive a COVID-19 vaccine [10]. The fact that the elderly are more adversely affected by the clinical results of COVID-19 disease and that the elderly mortality rate is greater with respect to young people may explain the higher vaccination rates in this group.

Male gender was associated with higher COVID-19 vaccine acceptance rates in most of the studies included in a systematic review exploring the COVID-19 vaccine hesitancy in healthcare workers [9]. In a cross-sectional study conducted of healthcare professionals in Turkey, it was reported that the desire to be vaccinated was lower in women compared to men. [10]. As for our study, there was no significant difference between the vaccinated and unvaccinated groups on assessment in terms of gender.

It was determined in our study that marital status and having a child were not a factor for receiving a COVID-19 vaccine. In a study conducted in China, consistent with our study, marital status or having a child did not affect COVID-19 vaccine acceptance [11]. Another similar study observed that having a child did not make any difference in terms of receiving a COVID-19 vaccine [12]. However, in a study performed in Turkey, the rate of those willing to have a COVID-19 vaccine was revealed to be significantly higher among married participants compared to single ones [10].

In review of the literature, it was recognized that education level and occupational status are amongst the factors that positively impact vaccine acceptance. For instance, it was observed that vaccine acceptance increased with rising education levels in a study published in the USA [13]. Similarly, on examination of the data from all our personnel in our study the vaccination rate of the participants with master/doctoral graduates was higher than those with other education levels except primary school graduates. Furthermore, in our study, nurses/midwives, administrative staff, patient attendants/cleaners and security guards/employees in the other category had a higher risk of not getting vaccinated/late vaccination compared to faculty members and doctors. Similarly, in other studies conducted abroad, it was demonstrated that the willingness of doctors to be vaccinated was higher than that of other healthcare workers [14,15]. In a study of 1720 people conducted in the Asia–Pacific region in February 2021, no difference was found between educational status and vaccine acceptance [16]. Although there are studies in the literature with revelation of no difference between education level and vaccine acceptance, the higher rates of vaccination amongst doctors in comparison to other healthcare workers may also be explained by both education level and their clinical research characteristics. This situation leads to the thought that it may have enabled them to obtain more information from scientific sources related to the favorable effects of the vaccines and to increase vaccine acceptance.

A study of healthcare workers in Egypt revealed that caring for COVID-19 patients was an important factor for the decision to vaccinate against COVID-19 [17]. In a systematic review exploring COVID-19 vaccine hesitancy in healthcare workers, direct contact/care with COVID-19 patients or higher perceived risk and fear of being infected with COVID-19 were associated with lower COVID-19 vaccination hesitancy in more than half of the studies [9]. Distinctively, another study, assessing vaccine acceptance of healthcare personnel, identified no correlation between COVID-19 vaccine acceptance and having a work role involving direct patient interaction [18]. As for our study, there was also no significant impact of working in the departments providing one-to-one care to a COVID-19 patient on the decision to have a COVID-19 vaccine. However, since providing direct care to patients contains risks for both patients and staff in terms of acquiring COVID-19 infection, we believe that organizing trainings/seminars periodically where accurate information about the vaccine is given may increase vaccine acceptance of this group.

Acquiring the COVID-19 infection was described as one of the reasons for not receiving the vaccine in a study conducted on healthcare workers working in an emergency department [19]. In another study done in Turkey, people suffering from COVID-19 had less consideration of receiving the vaccine in comparison to those who did not contract the disease [10]. In two studies performed in Egypt and Palestine, no difference was detected between having COVID-19 and the thought of getting vaccinated [14,17]. Similar to the results of the studies carried out in the USA and Turkey, it was demonstrated in our study that having COVID-19 increased the rate of not receiving a vaccine or delaying it by 2.36 times. During the period of our study, the right to vaccination was defined for healthcare workers at least one month following COVID infection in our country. It was observed that previous acquisition of COVID-19 was a reason for not vaccinating within the vaccinated and nonvaccinated groups. This may result from the view that immunity is still maintained due to previous infection. Another reason may be that those who suffered from mild disease are not as afraid of this infection as those who did not acquire the disease. Therefore, they may not have planned to vaccinate as a priority.

Our study showed that previous and current health perceptions did not affect COVID-19 vaccine acceptance. Similarly, other studies conducted in China by applying the SF-12 scale, and conducted in USA, no difference was encountered in the group considering vaccination and the hesitant group [11,18]. On examination of the studies evaluating the effect of having a chronic disease on vaccine acceptance, a review article examining COVID-19 vaccine hesitancy in healthcare workers, having a chronic illness was associated with a higher rate of COVID-19 vaccine acceptance [9]. It was also observed in a cross-sectional study performed in Turkey that individuals with chronic diseases were more willing to be vaccinated against COVID-19 [10]. Similarly, in our study, the proportion of participants with chronic diseases within the vaccinated group was shown to be significantly higher than the unvaccinated group, although multivariate analysis did not support this finding.

In our study, it was observed that those who did not receive a flu vaccine this year had an inclination to not receive a COVID-19 vaccine (OR: 3.24). A study involving healthcare workers and the general population presented that people vaccinated against seasonal influenza have a strong tendency to accept a future COVID-19 vaccine [12]. In a study conducted on healthcare workers in France, it was demonstrated that vaccination against influenza in the previous season was the main predictor of COVID-19 vaccine acceptance [15]. Additionally, in a study carried out in London, the strongest predictor of vaccine acceptance was shown to be influenza vaccination acceptance [20]. As for a cross-sectional study performed in Turkey, whilst healthcare workers previously vaccinated against influenza were more willing to be vaccinated against COVID-19, no significant difference was found between those receiving tetanus and pneumococcal vaccines and those who did not in terms of willingness to get a COVID-19 vaccine [21]. Although the influenza vaccine is not mandatory in our country, it is recommended to be administered to healthcare workers every year. People receiving the influenza vaccine or other nonessential vaccines may have a higher risk perception and/or may be in the risk group, do more research on vaccines, or tend to have more healthy behaviors. For the same reasons, this may also explain why these individuals are inclined to have the COVID-19 vaccine.

In our study, univariate analysis showed that the rate of having one of the hepatitis A, hepatitis B or tetanus vaccines in the group immunizing with the COVID-19 vaccine was significantly higher than the group who refused the vaccine. Similar to our study, in a multicenter survey study exploring the hesitancy of taking the COVID-19 vaccine among healthcare workers in France, the rate of getting hepatitis B, mixed (diphtheria, tetanus, poliomyelitis) and pertussis vaccines was revealed to be lower in those who were less likely or unwilling to be vaccinated. [22]. In a cross-sectional study conducted in Egypt, people who received nonmandatory vaccines were more likely to agree to have a COVID-19 vaccine than those who did not [17]. In our study, however, there was no significant difference between the groups in terms of the rate of people who previously obtained a paid vaccine for themselves or their children.

It is an expected situation that people who are hesitant about other vaccines will also experience hesitancy about a newly produced vaccine such as a COVID-19 vaccine. In a study conducted on 2047 healthcare workers in France, it was detected that uncertainty toward other vaccines is an obstacle to having a COVID-19 vaccine (95% CI: 3.82–11.44) [15]. In our study, since the largest risk factor in COVID-19 vaccine hesitancy was hesitancy toward other vaccines; thus, eliminating the hesitancy related to other vaccines amongst healthcare personnel may help to increase the acceptance of COVID-19 vaccines. These individuals may have a greater impact than perceived in increasing vaccine hesitancy by misinformed people who do not have vaccine rejection but rather have reservations. Hence, providing education to these people regarding general vaccine hesitancy may improve acceptance for both the COVID-19 vaccine and other vaccines.

In our study, the three most common reasons for not accepting vaccination were doubt on vaccine effectiveness, lack of trust in its ingredients and fear of side effects. When we reviewed the literature, we also discovered these three reasons were among the most common causes of indecision. In another study conducted in the USA, the reason for delaying or rejecting the vaccine was evaluated; concerns regarding side effects, safety and efficacy were amongst the top three most common reasons [18]. Similarly, in another study investigating COVID-19 vaccine hesitancy in healthcare workers, these three reasons are the ones encountered most commonly [9]. For this reason, in order to remove vaccine hesitancy, it may be fundamentally beneficial to organize various educational activities with the aim of increasing the level of knowledge about the effect and the content of vaccines, and eliminating false information about the side effects. In addition, with the increase in the number of studies investigating the side-effect profile, it may be possible to evaluate more concretely whether this reservation is significant or not.

Two COVID-19 vaccines, both the inactive one and the one produced with mRNA technology, are administered in Turkey based on choice following March 31. In review of the 450 employees vaccinated after this date, the age of those who received the inactivated vaccine is higher. Whilst there was no difference in terms of gender, occupation and educational status, the rate of those working in a surgical branch was found to be greater in the mRNA vaccine group. Since 66% of the staff in our hospital was vaccinated in the first month, and there was only the inactive vaccine option available during that time, the group vaccinated after March 31 may not represent the general population. In addition, the third wave of COVID-19 disease in March and April throughout Turkey and the world may have affected people’s preferences. Our findings might differ if the right to choose had been given to everyone in the beginning.

### 4.1. Limitations

The first limitation of our study is that it was conducted within a single medical center. Concerning the time initiation of vaccination and performance of the survey, administration of a COVID-19 vaccine in the country was used less often worldwide, which may be recognized among the limitations of our study. Reaching all personnel was planned in the design of the questionnaire; however, this was not accomplished and may be considered a limitation of our study. Unvaccinated staff may have been more reluctant to participate in the study.

### 4.2. Strengths

Most of the studies in the literature investigating the tendency of healthcare workers to have the COVID-19 vaccine determine their attitudes toward a possible vaccine prior to the beginning of the vaccination process. Amongst its strengths, our study was conducted after vaccination took place and carried out in one of the country’s most important and largest hospitals. By both assessing all hospital staff and applying the questionnaire to the subgroups, an evaluation was made in two stages. In addition, it is one of our study’s strengths that the vaccination status of the personnel not receiving their vaccinations during the survey period was followed up on over time.

## 5. Conclusions

In order to increase acceptance, it is important to take steps addressing the root causes of existing hesitations. For this purpose, transparently sharing the information with the public regarding COVID-19 vaccine production processes and the results of vaccine administrations, including efficacy and safety data, may be important in enhancing the acceptance of vaccines. Furthermore, eliminating hesitancy related to other vaccines plays a key role in increasing COVID-19 vaccine acceptance. For this reason, providing general training regarding vaccines to healthcare workers, especially those under the age of 50 who are also found at risk in our study, the ones without the flu vaccine and the personnel indecisive about other vaccines both within hospitals and on a country basis, and repetitions of the training based on current data may be effective in improving vaccine acceptance. In order to increase vaccination rates amongst young people, giving messages directed towards the youth, the negative effects of the disease for themselves may be explained, thus enabling them to be role models for community immunity. The data concerning COVID-19 cases, patients and deaths are announced daily by the Ministry of Health. Stating the rates between those vaccinated and those who are not, via enabling the public to appreciate the effectiveness of vaccines concretely, will help to remove the distrust associated with the vaccine effectiveness, which is one of the main reasons for vaccine hesitancy.

## Figures and Tables

**Table 1:** Comparison of demographic characteristics concerning all personnel registered in the hospital according to their vaccination status.

	Vaccinated	Not Vaccinated	*p*-Value
	*n* = 3299	*n* = 638	
Age	38 (30–48) ***	33 (27–41) ***	<0.001 *
	*n* (%)	*n* (%)	
Gender			
Female	1861 (84.2%)	348 (15.8%)	0.385 **
Male	1438 (83.2%)	290 (16.8%)	
Education Status			
Primary School	241 (87%) ^a,b^	36 (13%) ^a,b^	<0.001 **
Secondary School	128 (78.5%) ^b,c^	35 (21.5%) ^b,c^	
High School	543 (77.6%) ^c^	157 (22.4%) ^c^	
University graduates	983 (80.4%) ^b,c^	239 (19.6%) ^b,c^	
Master graduate/Specialization/Doctorate	944 (90.5%) ^a^	101 (9.7%) ^a^	
Title			
Doctor	784 (91%) ^a^	78 (9%) ^a^	<0.001 **
Nurse	718 (78.1%) ^b^	201 (21.9%) ^b^	
Healthcare technician	335 (84%) ^b^	64 (16%) ^b^	
Administrative personnel–Security	528 (81.7%) ^b^	118 (18.3%) ^b^	
Cleaning personnel/Patient attendant	566 (82.4%) ^b^	121 (17.6%) ^b^	
Unit			
Basic–Laboratory	229 (91.6%) ^a^	21 (8.4%) ^a^	<0.001 **
Internal	942 (83.1%) ^b,c^	192 (16.9%) ^b,c^	
Surgical	1022 (84.5%) ^c^	187 (15.5%) ^c^	
Administrative–technical	725 (80%) ^b^	181(20%) ^b^	

Row percentages are given. * Mann–Whitney *U* test, ** chi-squared test, *** (median (25.–75.*p*)). For group analysis: each superscript letter denotes a subset of categories whose row value does not differ significantly from each other at the 0.05 level.

**Table 2:** Comparisons of vaccinated and unvaccinated healthcare personnel according to the survey.

Characteristics	Missing	Vaccinated Personnel	Unvaccinated Healthcare Personnel	*p*-Value
	%	*n* = 408 (%)	*n* = 297 (%)	
Age	6.5			
Under 50		313 (83.7%)	255 (89.5%)	0.033 *
Over 50		61 (16.3%)	30 (10.8%)	
Gender	0			
Female		228 (55.9%)	180 (60.6%)	0.210 **
Male		180 (44.1%)	117 (39.4%)	
Marital Status	2			
Married		238 (59.9%)	182 (61.9%)	0.603 **
Single\Widow		159 (40.1%)	112 (38.1%)	
Having a child	1.8			
No		199(50%)	131 (44.6%)	0.157 **
Yes		199 (50%)	163 (55.4%)	
Education	1.1			
Master or higher		172 (42.5%) ^a^	64 (21.9%) ^b^	<0.001 **
University		142 (35.1%) ^a^	139 (47.6%) ^b^	
High School		50 (12.3%) ^a^	57 (19.5%) ^b^	
Primary\Secondary School		41 (10.1%) ^a^	32 (11%) ^a^	
Occupation	0.1			
Faculty member\Specialist doctor/Resident doctor		142 (34.8%) ^a^	48 (16.2%) ^b^	<0.001 **
Nurse\Midwife		92 (22.5%) ^a^	63 (21.3%) ^a^	
Administrative personnel		50 (12.3%) ^a^	60 (20.3%) ^b^	
Patient attendant/cleaning staff		47 (11.5%) ^a^	53 (17.9%) ^b^	
Security guard/other		77 (18.9%) ^a^	72 (24.3%) ^b^	
Chronic disease	0			
Absent		277 (67.9%)	223 (75.1%)	0.038 **
Present		131 (32.1%)	74 (24.9%)	
Living with/taking care of someone at high risk for COVID-19 disease	1			
No		313 (77.7%)	220 (74.6%)	0.342 **
Yes		90 (22.3%)	75 (25.4%)	
Working in units that provide one-to-one care to COVID-19 patients	0.3			
No, I never worked		188 (46.2%) ^a^	160 (54.1%) ^b^	0.112 **
Yes, I am working		100 (24.6%) ^a^	65 (22%) ^a^	
I worked but no longer working		119 (29.2%) ^a^	72 (24%) ^a^	
Status of acquiring COVID-19	0.4			
No		332 (81.8%)	205 (69.3%)	<0.001 **
Yes		74 (18.2%)	91 (30.7%)	
Which of the following situations have you encountered with COVID-19 in your family or close environment?	1.3			
There was no COVID-19 positivity in my environment.		129 (29.7%)	92 (31.5%)	0.824 **
Hospital admission/ICU admission/Death		126 (31.2%)	92 (31.5%)	
Had mild disease, not requiring hospitalization		158 (39.1%)	108 (37%)	
How would you rate your health status?	0.1			
Very bad–bad		70 (17.2%)	59 (19.9%)	0.541 **
Average		192 (47.2%)	142 (47.8%)	
Good–perfect		145 (35.6%)	96 (32.3%)	
Compared to last year, what would you say about your current health status?	0.1			
Much worse–bad		73 (17.9%)	61 (20.5%)	0.304 **
Almost the same		280 (68.8%)	188 (63.3%)	
Good–much better		54 (13.3%)	48 (16.2%)	
Are you afraid of acquiring COVID-19 disease?	1.4			
No		182 (45.4%)	122 (41.5%)	0.307 **
Yes		219 (54.6%)	172 (58.5%)	
Have you received hepatitis A, hepatitis B or tetanus vaccinations?	0.9			
No		74 (18.3%)	78 (26.5%)	0.009 **
Yes		331 (81.7%)	216 (73.5%)	
Have you or your child had a paid vaccine before?	0.7			
No		273 (67.4%)	211 (71.5 %)	0.244 **
Yes		132 (32.6%)	84 (28.5%)	
Have you had the flu vaccine this year?	0.1			
No		314 (77.1%)	271 (91.2%)	<0.001 **
Yes		93 (22.9%)	26 (8.8%)	
Is there a hesitancy present in relation to any of the hesitancy questions about other vaccines?	0.4			
No		387 (94.9%)	207 (70.4%)	<0.001 **
Yes		21 (5.1%)	87 (29.6%)	
Have you ever refused a vaccine for yourself or your child because you thought it was dangerous or ineffective?	0.4			
No		396 (97.1%)	229 (77.9%)	<0.001 **
Yes		12 (2.9%)	65 (22.1%)	
Have you ever delayed a vaccination recommended by a doctor before?	0.1			
No		396 (97.1%)	259 (87.6%)	<0.001 **
Yes		12 (2.9%)	37 (12.5%)	

Column percentages are given * Mann–Whitney *U* test, ** chi-squared test. For group analysis: each superscript letter denotes a subset of categories whose column value do not differ significantly from each other at the 0.05 level.

**Table 3:** Evaluation of the variables that may have an impact on not getting vaccinated or its postponement, by using multivariate logistic regression analysis.

	Model 1			Model 2		
	O.R.	(95% CI)	*p*-Value	O.R.	(95% CI)	*p*-Value
Age group						
Over 50	Ref.		0.026	Ref.		0.032
Under 50	1.85	(1.08–3.18)		1.81	(1.05–3.10)	
Gender						
Female	Ref.		0.209	Ref.		0.198
Male	0.78			0.78		
Occupation						
Faculty member\Specialist doctor\Resident doctor	Ref.		<0.001	Ref.		<0.001
Nurse\Midwife	1.78	(1.05–3.01)		1.73	(1.02–2.93)	
Administrative personnel	3.42	(1.94–6.04)		3.32	(1.88–5.85)	
Patient attendant\Cleaning staff	4.11	(2.26–7.45)		3.95	(2.18–7.17)	
Security guard\other	2.96	(1.77–4.97)		2.83	(1.68–4.75)	
Status of acquiring COVID-19						
No	Ref.		<0.001	Ref.		<0.001
Yes	2.36	(1.57–3.54)		2.38	(1.58–3.57)	
Status of receiving the flu vaccine this year						
Yes	Ref.		<0.001	Ref.		<0.001
No	3.24	(1.90–5.55)		3.14	(1.84–5.35)	
Is there a hesitancy present in relation to any of the hesitancy questions about other vaccines?						
No	Ref.		<0.001			
Yes	6.61	(3.82–11.44)				
Have you ever refused a vaccine for yourself or your child because you thought it was dangerous or ineffective?						
No				Ref.		<0.001
Yes				7.09	(3.49–14.39)	
Have you ever delayed a vaccination recommended by a doctor before?						
No				Ref.		0.003
Yes				3.34	(1.52–7.34)	

Binary logistic regression analysis was applied. O.R.: odds ratio. Model 1—Nagelkerke R square: 0.275, Hosmer and Lemeshow: 0.845; Model 2—Nagelkerke R square: 0.275, Hosmer and Lemeshow 0.672.

## Data Availability

The datasets generated and/or analyzed during the current study are not publicly available due to Turkish Personal Data Protection Law no. 6698, but are available upon reasonable request to the corresponding author.
